# Editorial: Machine learning-driven insights into cognitive aging and behavioral changes

**DOI:** 10.3389/fragi.2026.1919954

**Published:** 2026-07-24

**Authors:** Jaiteg Singh, Amarjot Kaur Grewal, Sukhjit Singh Sehra, Sumeet Kaur Sehra

**Affiliations:** 1 Chitkara University, Rajpura, India; 2 Wilfrid Laurier University, Waterloo, ON, Canada; 3 University of California Merced, Merced, CA, United States; 4 Conestoga College, Kitchener, ON, Canada

**Keywords:** behavioral change, biomarkers, cognitive aging, machine learning, risk factors

## Introduction

Cognitive aging is a complex and multifaceted process, affecting memory, attention, executive function, and behavioral regulation throughout life (Murman, 2015). With the global population rapidly aging, it has become critical to understand the mechanisms that drive cognitive decline and behavioral change (Prince et al., 2024). Cognitive impairment and dementia are the main contributors to disability among older adults, placing substantial social and healthcare burdens around the world (Lovett et al., 2023).

Traditional approaches to studying cognitive aging have relied on clinical assessments, longitudinal cohort studies, and neuropsychological testing (Chen et al., 2023). While these methods have produced valuable insights, they often struggle to capture the complex and heterogeneous nature of cognitive decline across individuals. Increasingly, researchers are turning to machine learning (ML) methods to analyze large and diverse datasets, including neuroimaging data, physiological signals, behavioral measurements, and digital health records.

ML has been used to identify early indicators of mild cognitive impairment and dementia by combining behavioral, demographic, and biomarker data (Cavedoni et al., 2020). Challenges remain, particularly regarding model interpretability, generalization, and ethical use of health data. Integrating ML with cognitive and behavioral science frameworks is therefore essential to ensure meaningful insights.

This Research Topic focuses on ML’s potential to enhance the study of cognitive aging, including early detection of decline, behavioral patterns, and more personalized approaches to cognitive health.

## Overview of the contributing articles

The articles included in this Research Topic illustrate how ML methods can provide new insights into cognitive aging, behavioral change, and health outcomes in older populations. Although the studies differ in their data sources, populations, and analytical approaches, they collectively show how computational techniques can identify complex patterns that may be difficult to detect using conventional statistical methods.

One group of contributions focuses on “*physiological biomarkers and clinical risk prediction*”, demonstrating how ML can translate multidimensional health data into practical indicators of biological aging and adverse outcomes. For example, Periorbital skin index as a biomarker for biological aging and health status investigated the use of a periorbital skin index as a biomarker of biological aging and health status, highlighting the potential of ML-driven analysis of physiological features to provide non-invasive indicators of aging processes. Similarly, Cheng et al. developed a time-updated scoring system for predicting 3-month mortality among maintenance hemodialysis patients, illustrating how predictive modeling can support dynamic clinical risk assessment in older and medically vulnerable populations.

A second group examines “*cognitive decline and its associated risk factors*”, showing how ML can reveal heterogeneous and interacting determinants of cognitive impairment. In Xu et al., a comparative analysis of predictive models was conducted for cognitive dysfunction in patients with leukoaraiosis, demonstrating that different algorithms may capture distinct risk patterns associated with cognitive impairment. In a related study, Xu et al., automated ML methods were applied to identify factors associated with subjective cognitive decline, highlighting the capacity of data-driven models to uncover complex relationships among demographic, behavioral, and clinical variables.

A closely related theme concerns “*frailty and functional vulnerability*”, where predictive models can help connect cognitive status with broader care needs. In Ren et al., multiple models were developed and comparatively validated for predicting cognitive frailty among nursing-home residents. This contribution emphasizes the value of integrating ML into geriatric risk assessment and care planning, particularly in settings where cognitive and physical vulnerabilities frequently coexist.

Several contributions address “digital assessment and behavioral monitoring”, illustrating how observable signals from everyday performance can support earlier and less invasive detection of cognitive and functional change. In Koduma et al., the potential of an eye-tracking-based cognitive scale for screening mild cognitive impairment was investigated, demonstrating how digital behavioral signals may support the early identification of cognitive changes. Complementing this perspective, Jun et al. examined the relationship between activities of daily living and speech impediments using statistical and ML techniques, illustrating how communication patterns may reflect broader changes in functional independence.

Finally, the collection also considers “neural adaptation and compensatory mechanisms”, extending the role of computational analysis beyond the prediction of decline to the interpretation of resilience in the aging brain. In Solomons et al., right-hemisphere engagement during language processing was examined in older adults. The findings suggest that increased neural activity may reflect compensatory mechanisms rather than cognitive deterioration alone, highlighting how computational approaches can help distinguish adaptive reorganization from pathological decline.

Taken together, these contributions demonstrate the diverse ways in which ML can advance the study of cognitive aging. Across the themes of physiological measurement, cognitive risk prediction, frailty assessment, digital behavioral monitoring, and neural compensation, the studies show how computational methods can integrate complex and heterogeneous data into more informative models of aging. Such approaches may improve understanding of how cognitive function changes across later life while supporting the earlier identification of decline, vulnerability, and adaptive capacity.

## Outlook and future directions

The studies presented in this Research Topic highlight the growing role of ML in advancing research on cognitive aging and behavioral change. This Research Topic was launched to bring together computational, clinical, and behavioral perspectives on the detection, prediction, and interpretation of age-related cognitive change. Although the studies differ in their data sources, populations, and analytical approaches, they collectively show how computational techniques can identify complex patterns that may be difficult to detect using conventional statistical methods. As data collection technologies continue to expand, future research will likely integrate an increasingly diverse set of data sources, including neuroimaging data, wearable sensor measurements, electronic health records, and digital behavioral traces.

Another key area to explore in the future is the models’ interpretability. Although the ML system can be highly accurate in its predictions, its effectiveness in the real world depends on its reliability. Using explainable AI and feature attribution can help us understand the mechanisms behind the predictive results.

Ethical considerations, including data privacy, algorithmic bias, and equitable access to AI-driven healthcare technologies, must be carefully addressed.

By fostering collaboration across disciplines and integrating computational approaches with behavioral and clinical research, this collection has contributed to the development of more accurate, machine-interpretable, and impactful tools to understand and support cognitive health in aging populations.
